# Tolfenamic acid-induced alterations in genes and pathways in pancreatic cancer cells

**DOI:** 10.18632/oncotarget.14651

**Published:** 2017-01-14

**Authors:** Umesh T. Sankpal, Steve Goodison, Michelle Jones-Pauley, Myrna Hurtado, Fan Zhang, Riyaz Basha

**Affiliations:** ^1^ Texas College of Osteopathic Medicine, University of North Texas Health Science Center, TX, USA; ^2^ Department of Health Sciences Research, Mayo Clinic, Jacksonville, FL, USA; ^3^ Institute for Molecular Medicine, University of North Texas Health Science Center, TX, USA

**Keywords:** tolfenamic acid, pancreatic cancer, Sp1, microarray analysis

## Abstract

Non-steroidal anti-inflammatory drugs (NSAIDs) are being tested extensively for their role in the treatment and prevention of several cancers. Typically NSAIDs exhibit anti-tumor activities via modulation of cyclooxygenase (COX)-dependent mechanisms, however, an anti-cancer NSAID tolfenamic acid (TA) is believed to work through COX-independent pathways. Results from our laboratory and others have demonstrated the anti-cancer activity of TA in various cancer models including pancreatic cancer. TA has been shown to modulate certain cellular processes including, apoptosis, reactive oxygen species and signaling. In this study, molecular profiling was performed to precisely understand the mode of action of TA. Three pancreatic cancer cell lines, L3.6pl, MIA PaCa-2, and Panc1 were treated with TA (50 μM for 48 h) and the changes in gene expression was evaluated using the Affymetrix GeneChip Human Gene ST Array platform. Microarray results were further validated using quantitative PCR for seven genes altered by TA treatment in all three cell lines. Functional analysis of differentially expressed genes (2 fold increase or decrease, *p* < 0.05) using Ingenuity Pathway Analysis software, revealed that TA treatment predominantly affected the genes involved in cell cycle, cell growth and proliferation, and cell death and survival. Promoter analysis of the differentially expressed genes revealed that they are enriched for Sp1 binding sites, suggesting that Sp1 could be a major contributor in mediating the effect of TA. The gene expression studies identified new targets involved in TA's mode of action, while supporting the hypothesis about the association of Sp1 in TA mediated effects in pancreatic cancer.

## INTRODUCTION

Pancreatic cancer is an aggressive disease with poor prognosis and a median survival of 4–6 months. It is the fourth leading cause of cancer related deaths in the United States with a 5-year survival rate of about 5–6 % [1]. It remains undetected and asymptomatic in the initial stages, and is usually diagnosed when it reaches a metastatic stage, and surgery, chemotherapy and radiation have minimal effect on survival at this stage. Signaling, reactive oxygen species and transcription factors such as NF-kB are known to play critical role in pancreatic cancer [2–5]. Due to high aggressive nature of this malignancy, traditional chemotherapy is not often effective for treatment. The strategies involving combination treatment with plant derivatives showed effective response in some cancers [6] and such strategies are under investigation for pancreatic cancer [7]. Like many cancers, there is indication that inflammation plays an important part in pancreatic cancer initiation. There is also growing evidence demonstrating the role of non-steroidal anti-inflammatory drugs (NSAIDs) in cancer prevention and as anti-cancer agents. Meta-analysis and data from clinical trials have shown that NSAIDs, such as aspirin, can reduce the risk of certain cancers including pancreatic and colorectal cancer [8, 9]. The anti-cancer activity of NSAIDs is associated with the disruption of a variety of cellular processes such as cell cycle, apoptosis, and angiogenesis and can occur in a cyclooxygenase (COX)-dependent or COX-independent manner [10–13].

Studies from our laboratory and others using the NSAID tolfenamic acid (TA) have demonstrated its potential as an anti-cancer agent in several cancer models including pancreatic cancer [14–18]. We have also shown that TA acts as a chemopreventive agent [19]. The anti-cancer activity of TA is associated with the degradation of the specificity protein 1 (Sp1) transcription factor and the inhibition of expression of its downstream targets such as cMet, VEGF, and Survivin [15, 16, 18]. Unlike many other anti-cancer NSAIDs, TA works via a COX-independent mechanism and, accordingly, has a significantly reduced toxicity profile [20]. Decrease in Sp1 protein expression by TA treatment has been demonstrated in several cancer models, both *in vitro* and *in vivo* [14–16]. It has also been shown that TA treatment leads to cell cycle arrest, increase in apoptosis, and induction of ROS [15, 16, 21]. In this study we analyzed the gene expression profiles of three pancreatic cancer cell lines treated with 50 μM TA, with the aim of identifying genes or pathways affected by TA treatment.

RNA from three pancreatic cancer cell lines (L3.6pl, MIA PaCa-2, and Panc1) treated with physiologically relevant dose of TA (50 μM) was profiled to identify differential gene expression using human GeneChip 1.0 ST Arrays (Affimetrix). Classification of these genes using Ingenuity Pathway Analysis showed that they could be grouped into functional categories, specifically cell cycle, proliferation, and cell death. Promoter analysis of the differentially expressed genes identified an enrichment of Sp1 binding sites, suggesting that many of the identified genes could be regulated by the Sp1 transcription factor. This study corroborates our earlier findings and highlights the importance of targeting Sp1 and its downstream targets.

## RESULTS

### Genes expression regulation by TA treatment

**Figure 1:**
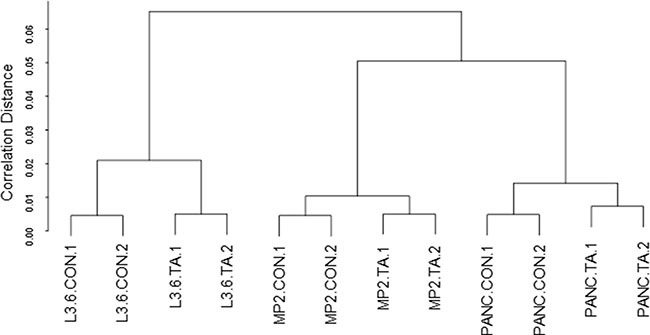
Hierarchical clustering Hierarchical cluster analysis using euclidean distance was performed to cluster genes and samples to generate a dendrogram.

Differential gene expression by TA treatment in three pancreatic cancer cell lines L3.6pl, MIA PaCa-2, and Panc1 was determined using Human GeneChip 1.0 ST array. Hierarchical clustering was performed using the Euclidian distance-generating function with probe sets differentially expressed in the microarray analysis. Our results showed that samples were distributed into two well-differentiated clusters (Figure 1). The first cluster contained samples from L3.6pl cells and the other cluster contained samples from MIA PaCa-2 and Panc1 cells. The dendrogram further branched into two groups; untreated and treated samples (Con and TA treated). The hierarchical clustering analysis suggests that distinct set of genes are upregulated by TA treatment in each of the three cell lines.

The number of differentially expressed genes (from a total of 28,869 genes represented on the GeneChip Array), for each of the three cell lines are shown in Table 1. A total of 957 (2601) genes were differentially expressed in L3.6pl, 318 (1317) genes in MIA PaCa-2, and 243 (1226) genes in Panc1 at fold change of ≥ 2 (or 1.5) and *p* < 0.05 by TA treatment for 48 h (Table 1). This data suggests that TA treatment had a greater effect on gene expression in L3.6pl cells compared to MIA PaCa-2 or Panc1 cells. This is also reflected in the cell viability data, where L3.6pl cells were more sensitive to TA treatment compared to the other two cell lines (data not shown).

**Table 1:** Differentially expressed genes in pancreatic cancer cell lines treated with TA

	L3.6pl		MIA PaCa-2		Panc1		Common	
	Up	Down	Up	Down	Up	Down	Up	Down
FC ≥ 2	253	704	211	107	106	137	21	14
FC ≥ 1.5	1058	1543	707	610	456	770	102	161

**Figure 2:**
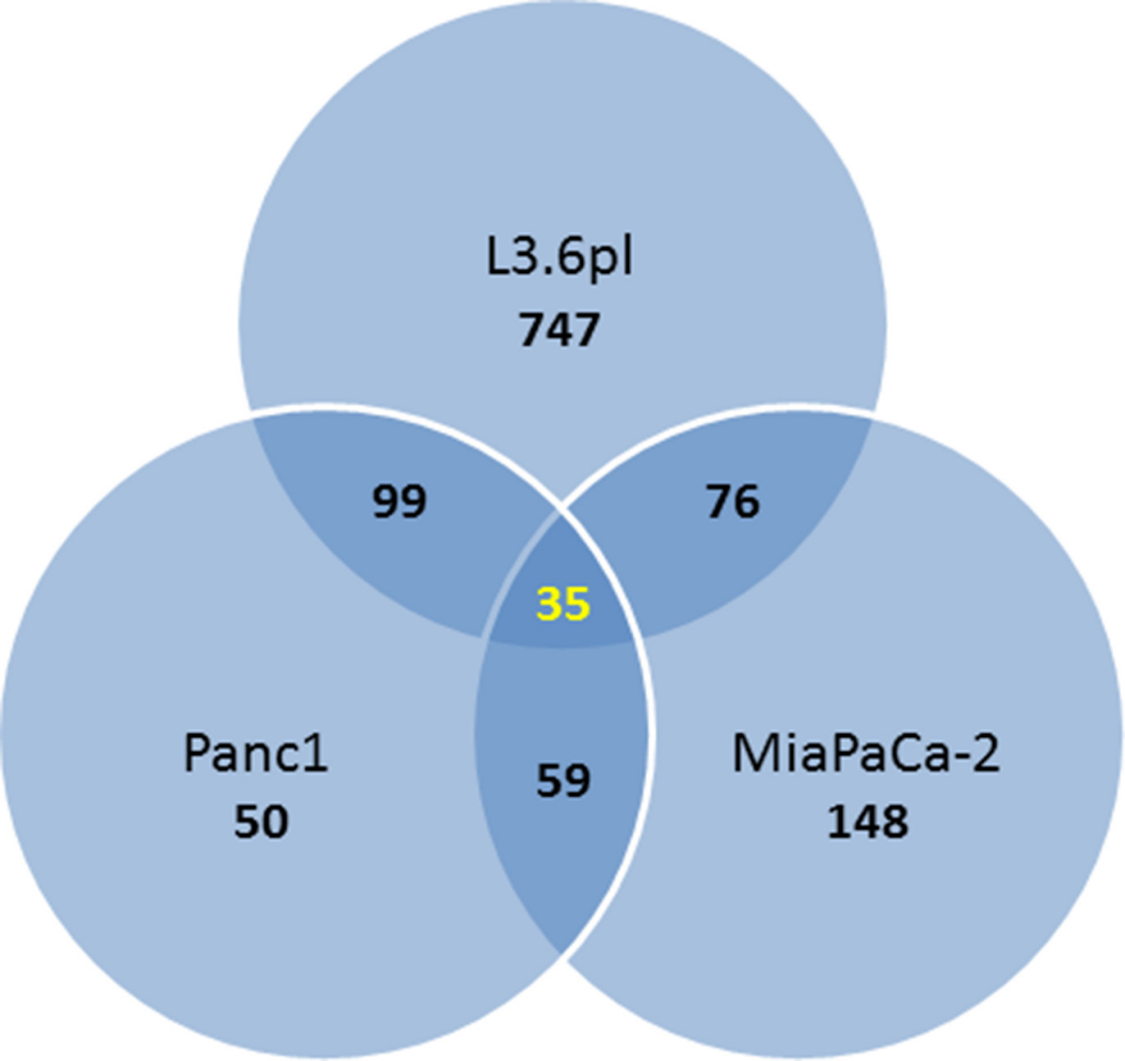
Venn diagram showing the number of overlapping differentially expressed genes (fold change ≥ 2; *p*-value ≤ 0.05) in the three pancreatic cancer cell lines treated with TA

The majority of the genes differentially expressed by TA treatment in L3.6pl cells were found to be downregulated (704 down vs. 253 up), while the majority of genes affected by TA in MIA PaCa-2 were upregulated (211 up vs. 107 down). In Panc1, slightly more genes were downregulated by TA treatment (137 down vs 106 up). A subset of 35 genes were differentially expressed (≥ 2.0) in all three cell lines (Venn diagram Figure 2), of which 21 were upregulated and 14 downregulated (Table 2).

**Table 2:** List of genes differentially expressed (fold change ≥ 2; p-value ≤ 0.05) by TA treatment in all three pancreatic cancer cell lines

Gene Symbol	mRNA - Description
**Down-regulated**	
ASPM	asp (abnormal spindle) homolog, microcephaly associated (Drosophila)
**CENPF**	centromere protein F (mitosin)
DLEU2	deleted in lymphocytic leukemia 2 (non-protein coding)
FAM111B	family with sequence similarity 111, member B
IFIT1	interferon-induced protein with tetratricopeptide repeats 1
IFITM1	interferon induced transmembrane protein 1 (9–27)
IQGAP2	IQ motif containing GTPase activating protein 2
KIF11	kinesin family member 11
**KIF20A**	kinesin family member 20A
**LMNB1**	lamin B1
**MYB**	v-myb myeloblastosis viral oncogene homolog (avian)
**SKP2**	S-phase kinase-associated protein 2 (p45)
TTK	TTK protein kinase
**Up-regulated**	
ARHGEF2	Rho/Rac guanine nucleotide exchange factor (GEF) 2
CHAC1	ChaC, cation transport regulator homolog 1 (E. coli)
**DDIT3**	DNA-damage-inducible transcript 3
FAM129A	family with sequence similarity 129, member A
GTPBP2	GTP binding protein 2
HBEGF	heparin-binding EGF-like growth factor
IFRD1	interferon-related developmental regulator 1
LCN2	lipocalin 2
LURAP1L	leucine rich adaptor protein 1 like
MXD1	MAX dimerization protein 1
PCLO	piccolo (presynaptic cytomatrix protein)
PLIN2	adipose differentiation-related protein
SDSL	serine dehydratase-like
SESN2	sestrin 2
SLC16A9	solute carrier family 16, member 9 (monocarboxylic acid transporter 9)
SLC6A9	solute carrier family 6 (neurotransmitter transporter, glycine), member 9
TMEM154	transmembrane protein 154
TRIB3	tribbles homolog 3 (Drosophila)
TRNAP24P	transfer RNA proline 24 (anticodon AGG) pseudogene
UPP1	uridine phosphorylase 1

### Validation of microarray data

**Figure 3:**
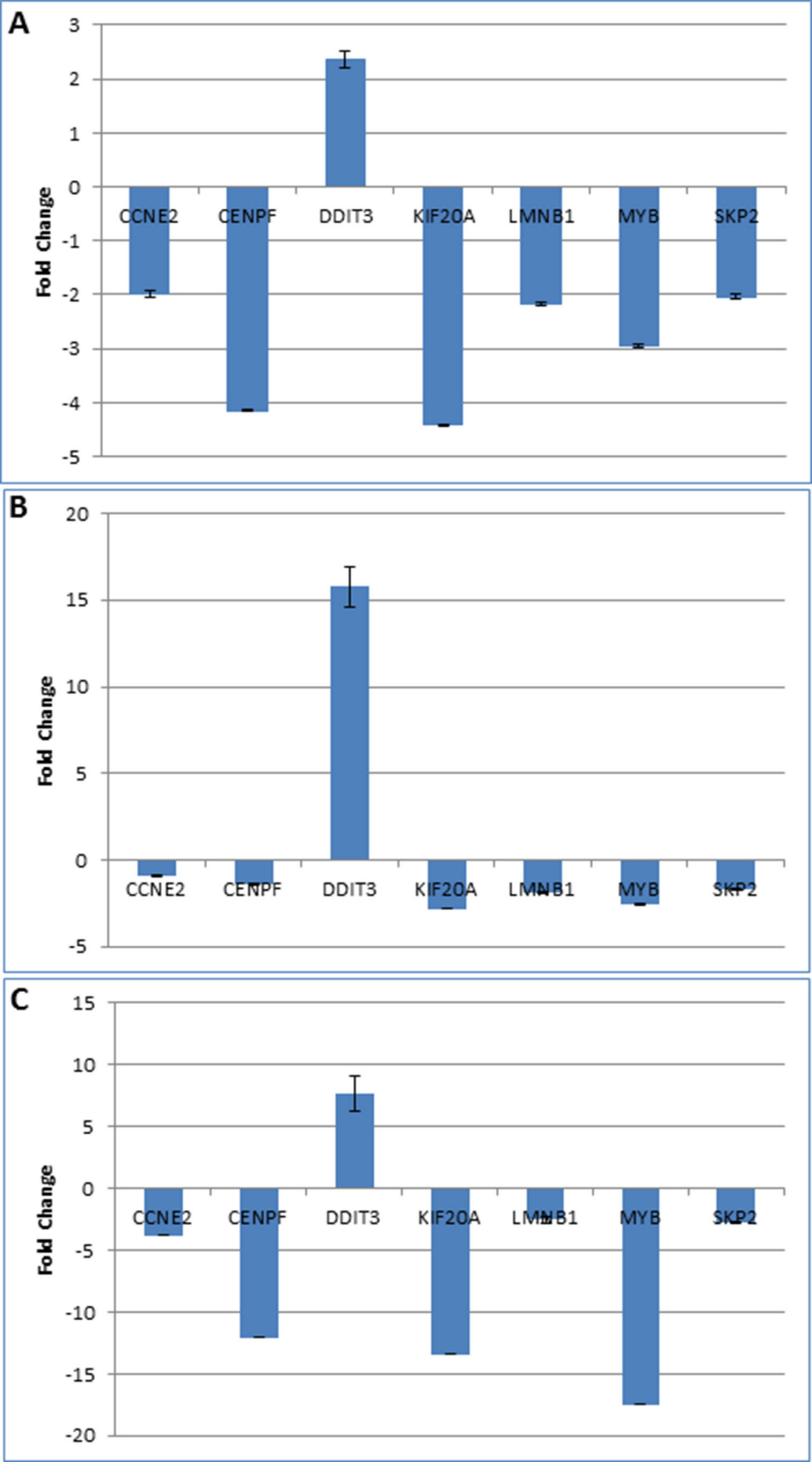
Validation of microarray results Quantitative PCR analysis was performed using TaqMan primer-probes for seven genes differentially expressed genes (selected from Table 2) in pancreatic cancer cell lines L3.6pl (**A**), MIA PaCa-2 (**B**), and Panc1 (**C**). Except for DDI3, which was upregulated, all other genes were downregulated by TA treatment. The figure shows the fold-change in gene expression in TA treated sample compared to DMSO treated control.

Gene expression changes, induced by TA and identified by the microarray analysis, were confirmed by quantitative PCR. Seven genes CENPF, KIF20A, LMNB1, MYB, SKP2, CCNE2, and DDIT3 were selected for validation studies (Table 2). Except for DDIT3, all other genes were downregulated by TA treatment. Also, six of these genes were differentially expressed in all three cells lines. CCNE2 was commonly expressed between L3.6pl and MIA PaCa-2 cell lines only. Quantitative PCR was carried out using TaqMan gene expression assay. As seen in Figure 3, the results of the qPCR analysis correlated with results from the microarray for all the three cell lines.

### Pathway analysis

Ingenuity pathway analysis (IPA) software was used to map the genes that were significantly affected by TA treatment in the three cell lines according to pathways, biological process, and disease classification. Also, relevant networks were explored using the IPA software. Table 3 shows the top five Molecular and Cellular functions to which the differentially expressed genes were mapped in the three cell lines. It is evident from the data that a set of genes related to Cell Cycle, Cell Growth and Proliferation, and Cell Death and Survival are altered by TA in all the three pancreatic cancer cell lines. Not surprisingly, cancer and gastrointestinal disease categories were in the top five networks affected by TA treatment (data not shown).

**Table 3:** Molecular and cellular functions associated with genes altered by TA treatment (fold change ≥ 2; p-value ≤ 0.05) in the three pancreatic cancer cell lines

Molecular and Cellular Functions	*p*-value	# Molecules
**L3.6pl**		
Cell Cycle	2.93E-37–1.67E-04	249
Cellular Assembly and Organization	4.44E-31–1.53E-04	188
DNA Replication, Recombination, and Repair	4.44E-31–1.38E-04	216
Cell Death and Survival	1.67E-21–1.59E-04	312
Cellular Growth and Proliferation	1.72E-18–1.32E-04	327
**MIA PaCa-2**		
Cellular Growth and Proliferation	6.28E-14–3.26E-04	130
Cell Death and Survival	5.07E-13–3.49E-04	112
Cellular Movement	8.63E-12–3.12E-04	69
Cell-To-Cell Signaling and Interaction	2.48E-09–3.26E-04	56
Cell Cycle	3.71E-09–3.17E-04	55
**Panc1**		
Cell Death and Survival	1.80E-12–3.77E-03	96
Cellular Growth and Proliferation	2.28E-10–3.77E-03	96
Cell Cycle	2.74E-09–3.77E-03	61
Cellular Assembly and Organization	2.21E-08–3.77E-03	51
DNA Replication, Recombination, and Repair	2.21E-08–2.15E-03	37
**Common Genes**		
Cell Death and Survival	2.22E-05–4.94E-02	14
Cellular Development	7.42E-05–4.94E-02	17
Cellular Growth and Proliferation	7.42E-05–4.94E-02	19
Cell Morphology	7.88E-05–4.89E-02	17
Cellular Function and Maintenance	7.88E-05–3.57E-02	14

### Promoter analysis

**Figure 4:**
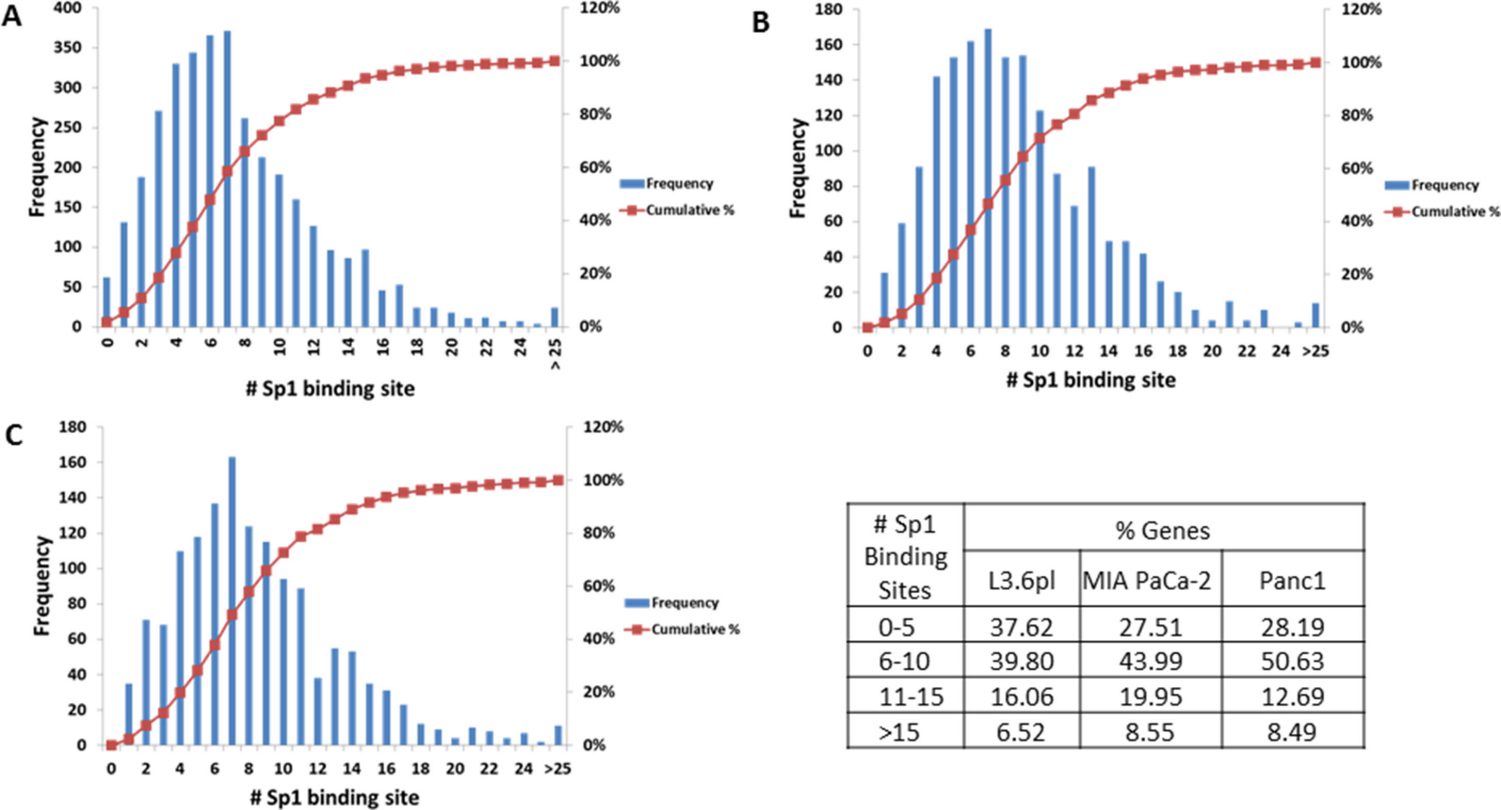
Promoter analysis Promoter sequences (1 kb upstream of transcription site) of the differentially expressed genes (fold change ≥ 1.5; *p*-value ≤ 0.05) were subject to analysis using the CLOVER program. The figure shows the number of putative Sp1 binding sites vs. the frequency of occurrence within the set of promoters analyzed for each cell line (**A**) L3.6pl, (**B**) MIA PaCa-2, and (**C**) Panc1. The data is summarized in the table which shows the percentage of genes with 0–5, 6–10, 11–15, and > 15 Sp1 binding sites in the three pancreatic cancer cell lines.

Previous studies with TA suggest that it targets the Sp1 transcription factor. To determine whether the differentially expressed genes by TA treatment are regulated by Sp1, promoter analysis was performed using the program Clover. Clover is an online resource that looks for enrichment of transcription factor binding sites. Promoter sequence (1 Kb upstream) for the genes differentially expressed by TA in each of the three cell lines was subject to analysis using Clover to determine the number of putative Sp1 binding sites. As seen in Figure 4, promoter analysis reveals that there are multiple Sp1 binding sites in the promoter regions of the genes, with 70–80% of the genes having at least ten Sp1 binding sites in all three cell lines.

## DISCUSSION

Prognosis for pancreatic cancer remains poor despite advances made in chemotherapies and surgical treatment modalities. The very low 5-year survival rate has not only prompted the search for novel treatment strategies but has also focused attention on chemoprevention as an alternative strategy. In this regard NSAIDs have recently been the focus of many studies as they possess both chemopreventive as well as anti-cancer properties [22]. Meta-analysis of population based studies provides evidence that long term use of NSAIDs (such as aspirin and ibuprofen) provide protection against the risk of developing certain cancers such as colon, pancreatic, breast, and prostate [23–25]. Since a majority of the NSAIDs target either the COX-1, or COX-2 enzymes, the mechanism of action underlying their anti-cancer and chemopreventive properties was thought to be mediated by COX-dependent mechanisms. However, there is growing evidence suggesting that COX-independent mechanisms also play an important role. For example, NSAIDs have been shown to regulate cell death and survival by targeting PPARδ, the NF-κB pathway, TGF-β, and the lipoxygenase pathway [26–28]. A study using microarray technology demonstrated that both non-selective and COX-2 selective NSAIDs induced apoptosis in colon cancer cells by activating death receptor pathway and the mitochondrial pathway [29]. A more recent study, also using microarray expression profiling, showed that anti-proliferative and anti-cancer activity of the NSAID ibuprofen results from the changes in expression of genes associated with biological oxidation, cell cycle, and apoptosis [30].

Our lab has been studying the NSAID tolfenamic acid (TA) as a potent anti-cancer agent. We and others have demonstrated its anti-cancer activity (both *in vitro* and *in vivo*) in several cancer models, including pancreatic, colon, lung, prostate, ovarian, and breast [14–17, 31, 32]. The anti-cancer activity of TA was shown to be associated with the degradation of the Sp (specificity protein) family of transcription factor and its downstream targets [18]. In pancreatic cancer, TA inhibited the proliferation of pancreatic cancer cells and tumors in mice [14]. This was associated with a decrease in Sp1 protein levels and its downstream targets VEGF and survivin [14, 18]. Sp1 belongs to the Sp family of transcription factors that includes Sp2, Sp3, and Sp4, all of which are shown to be involved in numerous biological processes [33, 34]. Sp1 has been shown to be upregulated in various cancers and is associated with poor prognosis [35–37]. It regulates genes associated with cell proliferation (cyclin D, cyclin E, Cdk2, E2F-1, c-Myc), metastasis (VEGF), and apoptosis (survivin, XIPA), all of which contribute to the hallmarks of cancer [33, 34]. These studies highlight the importance of Sp1 in cancer, thus making it an ideal target for therapy. Numerous compounds, including the NSAIDs such as TA, have been shown to target Sp1 and inhibit the expression of its downstream targets [34, 38].

In this study we carried out microarray based gene expression analysis to understand the mechanism underlying the anti-cancer activity of TA and identify potential new biomarkers. Our aim was to gain insights into pathways or processes, influenced by TA, that affect proliferation and induce apoptosis using a pancreatic cancer model. Our results indicate that the profile for the differentially expressed genes in the three pancreatic cancer cell lines was different (Figure 1). This is not surprising as our earlier results with cell proliferation showed that the three cell lines respond differently to TA treatment [18], with IC_50_ values of 42.3 μM for L3.6pl, 68.23 μM for MIA PaCa-2 and 57.50 μM for Panc1. L3.6pl had the most differentially expressed genes (fold change ≥ 2, *p*-value ≤ 0.05) compared to MIA PaCa-2 or Panc1 cells (Table 1). Pathway analysis was carried out using IPA software to determine the functional significance of the differentially expressed genes within the three cell lines. Our results indicate that TA treatment affects similar pathways in the three cell lines; ‘Cell Cycle’, ‘Cell Growth and Proliferation’, and ‘Cell Death and Survival’ (Table 3). This data correlates with similar microarray studies performed using NSAIDs, such as ibuprofen, NS-398, and indomethacin, which showed that these compounds also affect genes involved in cell cycle regulation, cell proliferation and apoptosis [29, 30, 39]. Our previous studies have shown that TA targets the degradation of the Sp1 transcription factor in pancreatic cancer cells [18]. A recent microarray study showed that knockdown of Sp1 using siRNA leads to alteration in genes related to Cell death and survival, and Cell growth and proliferation [40], similar to our observations with TA. This suggests that the differential gene expression by TA treatment is mediated via its effects on Sp1 transcription factor.

To determine if the differentially expressed genes in the three cell lines are regulated by Sp1 we analyzed the promoters of these genes for enrichment of transcription factor binding sites using the CLOVER program. Results indicate that there is enrichment for Sp1 binding sites within the promoters, strongly suggesting possible regulation of these genes by the Sp1 transcription factor (Figure 4). We were also able to validate the microarrays data using quantitative PCR using TaqMan probes for seven genes that are differentially expressed in all the three cell lines, CCNE2, CENPF, DDIT3, KIF20A, LMNB1, MYB, and SKP2 (Figure 3). There is evidence in the literature demonstrating the role of these TA targeted genes in cancer. CCNE2 (CyclinE2) is a cyclin that is required for G1/S transition and its expression is upregulated in tumor-derived cells [41]; CENPF (centromere protein F), is localized to the nuclear matrix in G2 phase and may play a role in chromosome segregation during mitosis, its expression is correlated to poor prognosis in prostate cancer patients [42]; DDIT3 (DNA damage inducible transcript 3), is activated by ER stress and it promotes apoptosis [43]; KIF20A (kinesin family member 20A), is a microtubulin-associated kinesin that is involved in migration and invasion of pancreatic cancer cells [44]; LMNB1 (lamin B1), is a component of the nuclear lamina and play a key role in nuclear structural integrity and chromosomal stability [45]; MYB (MYB proto-oncogene), functions as a transcription regulator and is involved in cell cycle, proliferation and migration [46]; SKP2 (S-phase kinase-associated protein 2), is an essential element of the cyclin A-CDK2 S-phase kinase and its expression correlates with metastasis and poor outcomes in pancreatic cancer patients [47]. We recognize that additional studies will be required to validate these gene products as potential biomarkers for TA's mode of action, and to further dissect the pivotal mechanisms involved in the anti-cancer activity of TA.

## MATERIALS AND METHODS

### Cell lines

MIA PaCa-2 and Panc1 human pancreatic carcinoma cells were obtained from American Type Culture Collection (ATCC. Manassas, VA). L3.6pl pancreatic cancer cells obtained from the MD Anderson Cancer Center (Houston, TX). The cell were maintained in Dulbecco's Modified Eagle Medium (DMEM) with high glucose (4500 mg/L) and supplemented with 5% fetal bovine serum, in a 37°C incubator with 5% CO_2_. All three cell lines were used for the microarray analysis and quantitative PCR.

### Treatment

Tolfenamic acid was purchased from Sigma-Aldrich (St. Louis, MO) and a 50 mM stock solution was prepared in DMSO. For treatment, cells were cultured overnight in a 100 mm dish. TA (50 μM) was added directly to the media and the cells cultured for additional 48 h. Control cells were treated with equivalent amounts of DMSO.

### Microarray analysis

MIA PaCa-2, Panc1, and L3.6pl cells were treated with 50 μM TA for 48 h. Total RNA was extracted from treated cells using TRIzol reagent. RNA was further purified using RNeasy mini kit (Qiagen, Germantown, MD). RNA yields were quantified using A260/280 and the quality assessed using an Agilent Bioanalyze (Agilent Technologies, Santa Clara, CA).

Microarray analysis was performed using Affymetrix GeneChip^®^ Human Gene 1.0 ST Arrays (Affymetrix, Santa Clara, CA) that consists of approximately 764,885 probe sets with a resolution number of 26 probes per gene, covering over 28,869 genes. The entire process was performed following the manufacturers’ instructions. Briefly, 500 ng of total RNA was used to synthesize double-stranded cDNA by a chimerical oligonucleotide with oligo-dT and T7 RNA polymerase. Biotin-labeled cRNA was prepared by linear amplification of the poly(A)^+^ RNA population within the total RNA sample. Exogenous positive controls were spiked into the total RNA before cDNA synthesis and were used to monitor the amplification and labeling process using a GeneChip^®^ Eukaryotic Poly-A RNA Control Kit (Affymetrix, Santa Clara, CA). The quantity and quality of the cRNA was assayed by spectrophotometry and using the Agilent Bioanalyzer. Biotinylated cRNA preparation was fragmented to uniform size and placed in hybridization cocktail containing biotinylated hybridization controls (GeneChip^®^ Expression Hybridization Controls, Affymetrix). Samples were hybridized onto a GeneChip^®^ Human Gene 1.0 ST Array at 45°C with 60 rpm for 17 hours in a Hybridization Oven 640 (Affymetrix, Santa Clara, CA). Microarray scanned images were obtained with a GeneChip Scanner 3000 7G (Affymetrix, Santa Clara, CA) using the default settings. Images were visually inspected to eliminate hybridization artifacts.

### Statistical analysis

Expression Console software (Affimetrix) was used to process the scanned images from arrays (gridding and feature intensity) and the data generated for each feature on the array were analyzed with GeneSpring software (Agilent Technologies, Santa Clara, CA). Raw intensity data for each gene on every array were normalized to the median intensity of the raw values from that array. Data for all arrays were filtered for intensity values that were above background in at least two of any set of three replicates for any condition within each drug treatment. To ensure that genes were reliably measured, ANOVA was used to compare the means of each condition (*n* = 3). Cutoff ratios > 2.0 and < 0.5 and *P* < 0.05 relative to the respective control group were selected for this study. Hierarchical cluster analysis using Euclidean distance was performed to cluster genes and samples to generate a heat map. Gene network and pathway analysis were performed using Ingenuity Pathway Analysis (http://www.ingenuity.com).

### Ingenuity pathway analysis

The functional significance of genes differentially expressed by TA treatment was evaluated using the Ingenuity Pathway Analysis (IPA) software (Ingenuity Systems version 6.3-1402). Genes with a fold change of > 1.5 and *P* < 0.05 were selected for network generation and pathway analyses implemented in IPA tools. GenBank IDs of the selected genes were uploaded into the IPA, which were mapped to the functional networks available in the Ingenuity Pathway Knowledge Base. Networks are composed of biological functions assigned to networks using significant *P*-values for focus gene functions compared with the whole Ingenuity Pathway Knowledge Base. Focus genes were identified as the subset having modeled interaction(s) with the other molecules in the database. A maximum of 35 molecules comprised a network. Each network was given a score reflecting the negative logarithm of the *P* value based on the chance of the significant molecules falling into the network by random. A score of 2 implies that there is a 1 in 100 chance that the focus genes are together in a network because of random chance. Therefore, scores of ≥ 2 have at least a 99% confidence of not being generated by random chance alone.

### Hierarchical clustering

Unsupervised clustering analysis was performed using a complete-linkage hierarchical clustering of a centered correlation similarity matrix with genes from the intrinsic gene list previously described. Genes were filtered and visualized, using the hclust function in R Package (V3.2.5).

### Real-time reverse transcription-PCR

Total RNA, extracted from control and TA treated cells was converted into single-stranded cDNA using Superscript III (Invitrogen, Carlsbad, CA). Quantitative PCR was performed with this cDNA using TaqMan gene expression assay for the selected genes and 96-well LightCycler 96 Real-Time PCR system (Roche, Pleasanton, CA). Each sample was analyzed in triplicate and GAPDH was used as endogenous control. The Assay ID's for the selected genes were, CENPF (Hs01118845_m1), KIF20A (Hs00993573_m1), LMNB1 (Hs01059210_m1), MYB (Hs00920556_m1), SKP2 (Hs01021864_m1), DDIT3 (Hs00358796_g1), and CCNE2 (Hs00180319_m1). The threshold cycle (C_T_) of the endogenous control was used to normalize target gene expression (ΔC_T_) to correct for experimental variation. The relative change in gene expression (ΔΔC_T_) was used to compare the gene expression in TA treated samples versus DMSO control. Gene expression results are presented as fold-change of TA treated sample with respect to DMSO control using the ΔΔC_T_ method. Differences between the groups were statistically evaluated by two-tailed paired *t* test. *P* < 0.05 was considered statistically significant. Presented data points represent an average ± SE of three experiments.

## References

[1] Hidalgo M (2010). Pancreatic cancer. N Engl J Med.

[2] Singh AP, Arora S, Bhardwaj A, Srivastava SK, Kadakia MP, Wang B, Grizzle WE, Owen LB, Singh S (2012). CXCL12/CXCR4 protein signaling axis induces sonic hedgehog expression in pancreatic cancer cells via extracellular regulated kinase- and Akt kinase-mediated activation of nuclear factor kappaB: implications for bidirectional tumor-stromal interactions. J Biol Chem.

[3] Arora S, Bhardwaj A, Singh S, Srivastava SK, McClellan S, Nirodi CS, Piazza GA, Grizzle WE, Owen LB, Singh AP (2013). An undesired effect of chemotherapy: gemcitabine promotes pancreatic cancer cell invasiveness through reactive oxygen species-dependent, nuclear factor kappaB- and hypoxia-inducible factor 1alpha-mediated up-regulation of CXCR4. J Biol Chem.

[4] Patel GK, Patton MC, Singh S, Khushman M, Singh AP (2016). Pancreatic Cancer Exosomes: Shedding Off for a Meaningful Journey. Pancreat Disord Ther.

[5] Tyagi N, Bhardwaj A, Singh AP, McClellan S, Carter JE, Singh S (2014). p-21 activated kinase 4 promotes proliferation and survival of pancreatic cancer cells through AKT- and ERK-dependent activation of NF-kappaB pathway. Oncotarget.

[6] Nessa MU, Beale P, Chan C, Yu JQ, Huq F (2012). Studies on combination of platinum drugs cisplatin and oxaliplatin with phytochemicals anethole and curcumin in ovarian tumour models. Anticancer Res.

[7] Basha R, Connelly SF, Sankpal UT, Nagaraju GP, Patel H, Vishwanatha JK, Shelake S, Tabor-Simecka L, Shoji M, Simecka JW, El-Rayes B (2016). Small molecule tolfenamic acid and dietary spice curcumin treatment enhances antiproliferative effect in pancreatic cancer cells via suppressing Sp1, disrupting NF-kB translocation to nucleus and cell cycle phase distribution. J Nutr Biochem.

[8] Bradley MC, Hughes CM, Cantwell MM, Napolitano G, Murray LJ (2010). Non-steroidal anti-inflammatory drugs and pancreatic cancer risk: a nested case-control study. Br J Cancer.

[9] Drew DA, Cao Y, Chan AT (2016). Aspirin and colorectal cancer: the promise of precision chemoprevention. Nat Rev Cancer.

[10] Gurpinar E, Grizzle WE, Piazza GA (2013). COX-Independent Mechanisms of Cancer Chemoprevention by Anti-Inflammatory Drugs. Front Oncol.

[11] Raut CP, Nawrocki S, Lashinger LM, Davis DW, Khanbolooki S, Xiong H, Ellis LM, McConkey DJ (2004). Celecoxib inhibits angiogenesis by inducing endothelial cell apoptosis in human pancreatic tumor xenografts. Cancer Biol Ther.

[12] Piazza GA, Keeton AB, Tinsley HN, Whitt JD, Gary BD, Mathew B, Singh R, Grizzle WE, Reynolds RC (2010). NSAIDs. Old Drugs Reveal New Anticancer Targets. Pharmaceuticals (Basel).

[13] Maier TJ, Schilling K, Schmidt R, Geisslinger G, Grosch S (2004). Cyclooxygenase-2 (COX-2)-dependent and -independent anticarcinogenic effects of celecoxib in human colon carcinoma cells. Biochem Pharmacol.

[14] Konduri S, Colon J, Baker CH, Safe S, Abbruzzese JL, Abudayyeh A, Basha MR, Abdelrahim M (2009). Tolfenamic acid enhances pancreatic cancer cell and tumor response to radiation therapy by inhibiting survivin protein expression. Mol Cancer Ther.

[15] Colon J, Basha MR, Madero-Visbal R, Konduri S, Baker CH, Herrera LJ, Safe S, Sheikh-Hamad D, Abudayyeh A, Alvarado B, Abdelrahim M (2011). Tolfenamic acid decreases c-Met expression through Sp proteins degradation and inhibits lung cancer cells growth and tumor formation in orthotopic mice. Invest New Drugs.

[16] Sankpal UT, Abdelrahim M, Connelly SF, Lee CM, Madero-Visbal R, Colon J, Smith J, Safe S, Maliakal P, Basha R (2012). Small molecule tolfenamic acid inhibits PC-3 cell proliferation and invasion in vitro, and tumor growth in orthotopic mouse model for prostate cancer. Prostate.

[17] Basha R, Ingersoll SB, Sankpal UT, Ahmad S, Baker CH, Edwards JR, Holloway RW, Kaja S, Abdelrahim M (2011). Tolfenamic acid inhibits ovarian cancer cell growth and decreases the expression of c-Met and survivin through suppressing specificity protein transcription factors. Gynecol Oncol.

[18] Abdelrahim M, Baker CH, Abbruzzese JL, Safe S (2006). Tolfenamic acid and pancreatic cancer growth, angiogenesis, and Sp protein degradation. J Natl Cancer Inst.

[19] Maliakal P, Abdelrahim M, Sankpal UT, Maliakal C, Baker CH, Safe S, Herrera LJ, Abudayyeh A, Kaja S, Basha R (2012). Chemopreventive effects of tolfenamic acid against esophageal tumorigenesis in rats. Invest New Drugs.

[20] Sankpal UT, Lee CM, Connelly SF, Kayaleh O, Eslin D, Sutphin R, Goodison S, Adwan L, Zawia NH, Lichtenberger LM, Basha R (2013). Cellular and organismal toxicity of the anti-cancer small molecule, tolfenamic acid: a pre-clinical evaluation. Cell Physiol Biochem.

[21] Sankpal UT, Nagaraju GP, Gottipolu SR, Hurtado M, Jordan CG, Simecka JW, Shoji M, El-Rayes B, Basha R (2016). Combination of tolfenamic acid and curcumin induces colon cancer cell growth inhibition through modulating specific transcription factors and reactive oxygen species. Oncotarget.

[22] Chan AT, Detering E (2013). An emerging role for anti-inflammatory agents for chemoprevention. Recent Results Cancer Res.

[23] Cuzick J, Otto F, Baron JA, Brown PH, Burn J, Greenwald P, Jankowski J, C La Vecchia, Meyskens F, Senn HJ, Thun M (2009). Aspirin and non-steroidal anti-inflammatory drugs for cancer prevention: an international consensus statement. Lancet Oncol.

[24] Harris RE, Beebe-Donk J, Doss H, Burr Doss D (2005). Aspirin, ibuprofen, and other non-steroidal anti-inflammatory drugs in cancer prevention: a critical review of non-selective COX-2 blockade (review). Oncol Rep.

[25] Tsioulias GJ, Go MF, Rigas B (2015). NSAIDs and Colorectal Cancer Control: Promise and Challenges. Curr Pharmacol Rep.

[26] Shureiqi I, Chen D, Lotan R, Yang P, Newman RA, Fischer SM, Lippman SM (2000). 15-Lipoxygenase-1 mediates nonsteroidal anti-inflammatory drug-induced apoptosis independently of cyclooxygenase-2 in colon cancer cells. Cancer Res.

[27] Mladenova D, Pangon L, Currey N, Ng I, Musgrove EA, Grey ST, Kohonen-Corish MR (2013). Sulindac activates NF-kappaB signaling in colon cancer cells. Cell Commun Signal.

[28] Baek SJ, Kim KS, Nixon JB, Wilson LC, Eling TE (2001). Cyclooxygenase inhibitors regulate the expression of a TGF-beta superfamily member that has proapoptotic and antitumorigenic activities. Mol Pharmacol.

[29] Huang RH, Chai J, Tarnawski AS (2006). Identification of specific genes and pathways involved in NSAIDs-induced apoptosis of human colon cancer cells. World J Gastroenterol.

[30] Bonelli P, Tuccillo FM, Calemma R, Pezzetti F, Borrelli A, Martinelli R, De Rosa A, Esposito D, Palaia R, Castello G (2011). Changes in the gene expression profile of gastric cancer cells in response to ibuprofen: a gene pathway analysis. Pharmacogenomics J.

[31] Liu X, Abdelrahim M, Abudayyeh A, Lei P, Safe S (2009). The nonsteroidal anti-inflammatory drug tolfenamic acid inhibits BT474 and SKBR3 breast cancer cell and tumor growth by repressing erbB2 expression. Mol Cancer Ther.

[32] Pathi S, Li X, Safe S (2014). Tolfenamic acid inhibits colon cancer cell and tumor growth and induces degradation of specificity protein (Sp) transcription factors. Mol Carcinog.

[33] Beishline K, Azizkhan-Clifford J (2015). Sp1 and the ‘hallmarks of cancer’. FEBS J.

[34] Vizcaino C, Mansilla S, Portugal J (2015). Sp1 transcription factor: A long-standing target in cancer chemotherapy. Pharmacol Ther.

[35] Wang J, Kang M, Qin YT, Wei ZX, Xiao JJ, Wang RS (2015). Sp1 is over-expressed in nasopharyngeal cancer and is a poor prognostic indicator for patients receiving radiotherapy. Int J Clin Exp Pathol.

[36] Yao JC, Wang L, Wei D, Gong W, Hassan M, Wu TT, Mansfield P, Ajani J, Xie K (2004). Association between expression of transcription factor Sp1 and increased vascular endothelial growth factor expression, advanced stage, and poor survival in patients with resected gastric cancer. Clin Cancer Res.

[37] Guan H, Cai J, Zhang N, Wu J, Yuan J, Li J, Li M (2012). Sp1 is upregulated in human glioma, promotes MMP-2-mediated cell invasion and predicts poor clinical outcome. Int J Cancer.

[38] Sankpal UT, Maliakal P, Bose D, Kayaleh O, Buchholz D, Basha R (2012). Expression of specificity protein transcription factors in pancreatic cancer and their association in prognosis and therapy. Curr Med Chem.

[39] John-Aryankalayil M, Palayoor ST, Cerna D, Falduto MT, Magnuson SR, Coleman CN (2009). NS-398, ibuprofen, and cyclooxygenase-2 RNA interference produce significantly different gene expression profiles in prostate cancer cells. Mol Cancer Ther.

[40] Hedrick E, Cheng Y, Jin UH, Kim K, Safe S (2016). Specificity protein (Sp) transcription factors Sp1, Sp3 and Sp4 are non-oncogene addiction genes in cancer cells. Oncotarget.

[41] Zhao GH, Xie GQ, Miao YY (2012). [Expression and clinical significance of cyclin E2 in nasopharyngeal carcinoma]. Xi Bao Yu Fen Zi Mian Yi Xue Za Zhi.

[42] Zhuo YJ, Xi M, Wan YP, Hua W, Liu YL, Wan S, Zhou YL, Luo HW, Wu SL, Zhong WD, Wu CL (2015). Enhanced expression of centromere protein F predicts clinical progression and prognosis in patients with prostate cancer. Int J Mol Med.

[43] Namba T, Kodama R (2015). Avarol induces apoptosis in pancreatic ductal adenocarcinoma cells by activating PERK-eIF2alpha-CHOP signaling. Mar Drugs.

[44] Stangel D, Erkan M, Buchholz M, Gress T, Michalski C, Raulefs S, Friess H, Kleeff J (2015). Kif20a inhibition reduces migration and invasion of pancreatic cancer cells. J Surg Res.

[45] Wazir U, Ahmed MH, Bridger JM, Harvey A, Jiang WG, Sharma AK, Mokbel K (2013). The clinicopathological significance of lamin A/C, lamin B1 and lamin B receptor mRNA expression in human breast cancer. Cell Mol Biol Lett.

[46] Srivastava SK, Bhardwaj A, Arora S, Singh S, Azim S, Tyagi N, Carter JE, Wang B, Singh AP (2015). MYB is a novel regulator of pancreatic tumour growth and metastasis. Br J Cancer.

[47] Einama T, Kagata Y, Tsuda H, Morita D, Ogata S, Ueda S, Takigawa T, Kawarabayashi N, Fukatsu K, Sugiura Y, Matsubara O, Hatsuse K (2006). High-level Skp2 expression in pancreatic ductal adenocarcinoma: correlation with the extent of lymph node metastasis, higher histological grade, and poorer patient outcome. Pancreas.

